# Association of immune cell composition with the risk factors and incidence of acute coronary syndrome

**DOI:** 10.1186/s13148-023-01527-4

**Published:** 2023-07-17

**Authors:** Xian Shi, Minghan Qu, Yi Jiang, Ziwei Zhu, Chengguqiu Dai, Minghui Jiang, Lin Ding, Yu Yan, Chaolong Wang, Xiaomin Zhang, Shanshan Cheng, Xingjie Hao

**Affiliations:** 1 Department of Epidemiology and Biostatistics, Ministry of Education Key Laboratory of Environment and Health and State Key Laboratory of Environmental Health (Incubating), School of Public Health, Tongji Medical College, Huazhong University of Science and Technology, Wuhan, China; 2 Department of Occupational and Environmental Health, Ministry of Education Key Laboratory of Environment and Health and State Key Laboratory of Environmental Health (Incubating), School of Public Health, Tongji Medical College, Huazhong University of Science and Technology, Wuhan, China

**Keywords:** Acute coronary syndrome, Immune cell composition, Risk factor, DNA methylation

## Abstract

**Background:**

Although immune cells are involved in acute coronary syndrome (ACS), few studies have explored the association of incident ACS with the relative immune cell proportions. We aimed to investigate the association of immune cell proportions with the incidence and risk factors of ACS in the Dongfeng–Tongji cohort.

**Methods:**

We conducted the analyses with 38,295 subjects from the first follow-up of the Dongfeng–Tongji cohort, including DNA methylation profiles for 1570 individuals. The proportions of immune cell types were observed from routine blood tests or estimated from DNA methylation profiles. For both observed and estimated immune cell proportions, we tested their associations with risk factors of ACS by multivariable linear regression models. In addition, the association of each immune cell proportion with incident ACS was assessed by the Cox regression model and conditional logistic regression model, respectively, adjusting for the risk factors of ACS.

**Findings:**

The proportions of lymphocytes, monocytes, and neutrophils showed strong associations with sex, followed by diabetes. Moreover, sex and current smoking were the two factors with strongest association with the proportions of lymphocyte subtypes. The hazard ratio (HR) and 95% confidence interval (CI) of incident ACS per standard deviation (SD) increase in proportions of lymphocytes and neutrophils were 0.91 (0.85–0.96) and 1.10 (1.03–1.16), respectively. Furthermore, the OR (95% CI) of incident ACS per SD increase in proportions of NK cells, CD4^+^ T cells, and B cells were 0.88 (0.78–0.99), 1.15 (1.03–1.30), and 1.13 (1.00–1.26), respectively.

**Interpretation:**

The proportions of immune cells were associated with several risk factors of ACS, including sex, diabetes, and current smoking. In addition, proportion of neutrophils had a risk effect, while proportion of lymphocytes had a protective effect on the incidence of ACS. The protective effect of lymphocytes was probably driven by NK cells.

**Supplementary Information:**

The online version contains supplementary material available at 10.1186/s13148-023-01527-4.

## Introduction

Acute coronary syndrome (ACS) includes unstable angina and myocardial infarction (MI), with the underlying mechanism involving atherosclerotic plaque rupture or erosion followed by coronary thrombosis [1]. ACS is an acute manifest of ischemic heart disease (IHD) with a high mortality rate, accounting for substantial disease burden and nearly half of all deaths from IHD [2, 3]. Previous studies have shown that ACS was associated with several common risk factors, such as sex, age, smoking, obesity, hypertension, dyslipidemia, and diabetes [4, 5]. In addition, immune cells are associated with atherosclerosis [6–8], and immune cell-related indicators, such as white blood cell counts (WBC), neutrophil counts, and neutrophil-to-lymphocyte ratio (NLR), were associated with the incidence of ACS [9, 10]. However, they did not evaluate the proportions of main leukocyte subtypes, nor did they assess the proportions of lymphocyte subtypes due to the limitations of routine blood tests. Understanding the association between the proportions of main leukocyte subtypes and lymphocyte subtypes and the incidence of ACS might help to discover new biomarkers for better assessment of the risk of ACS.

In addition to ACS, risk factors of ACS were also associated with the immune cell proportions [11–19], but inconsistent conclusions were reported by different studies. For example, Chen et al. [11] identified a lower percentage of lymphocytes in adult males compared to females, while such association was not supported by Wongkrajang et al. [12]. Moreover, studies have found that sex differences in lymphocyte percentage were reversed in middle-aged adults [20, 21]. A few studies identified a lower percentage of lymphocytes in current smokers in Europeans [13, 22], whereas Tollerud et al. [15] showed an opposite association in African-Americans. Furthermore, Akesson et al. [18] reported a negative association between the relative abundance of NK cells and diabetes, whereas Lv et al. [19] reported a positive association. Inconsistent conclusions across studies were probably due to potential unadjusted confounders [11, 12, 18, 19], small sample sizes [12, 14, 18, 19, 23], or different ethnic backgrounds [13, 15, 22]. Therefore, joint analysis of the associations between these risk factors and immune cell composition in a large sample might help resolve the inconsistency.

Routine blood tests are the most convenient method for measuring main leukocyte subtypes, however, they can not enable further analysis of the lymphocyte subtypes. With the availability of DNA methylation profiles, several algorithms have been developed to estimate the proportions of lymphocyte subtypes [24–26]. Therefore, by leveraging the proportions of immune cells observed from routine blood tests and those estimated from DNA methylation, we proposed to investigate the association of immune cell composition with the incidence and risk factors of ACS. We found that the proportions of immune cells were significantly associated with sex, current smoking, and diabetes. In addition, the proportions of neutrophils, lymphocytes, and NK cells were associated with the incidence of ACS. The homeostasis of immune cell composition is associated with common risk factors and the incidence of ACS.

## Materials and methods

### Phenotype data

**Fig. 1:**
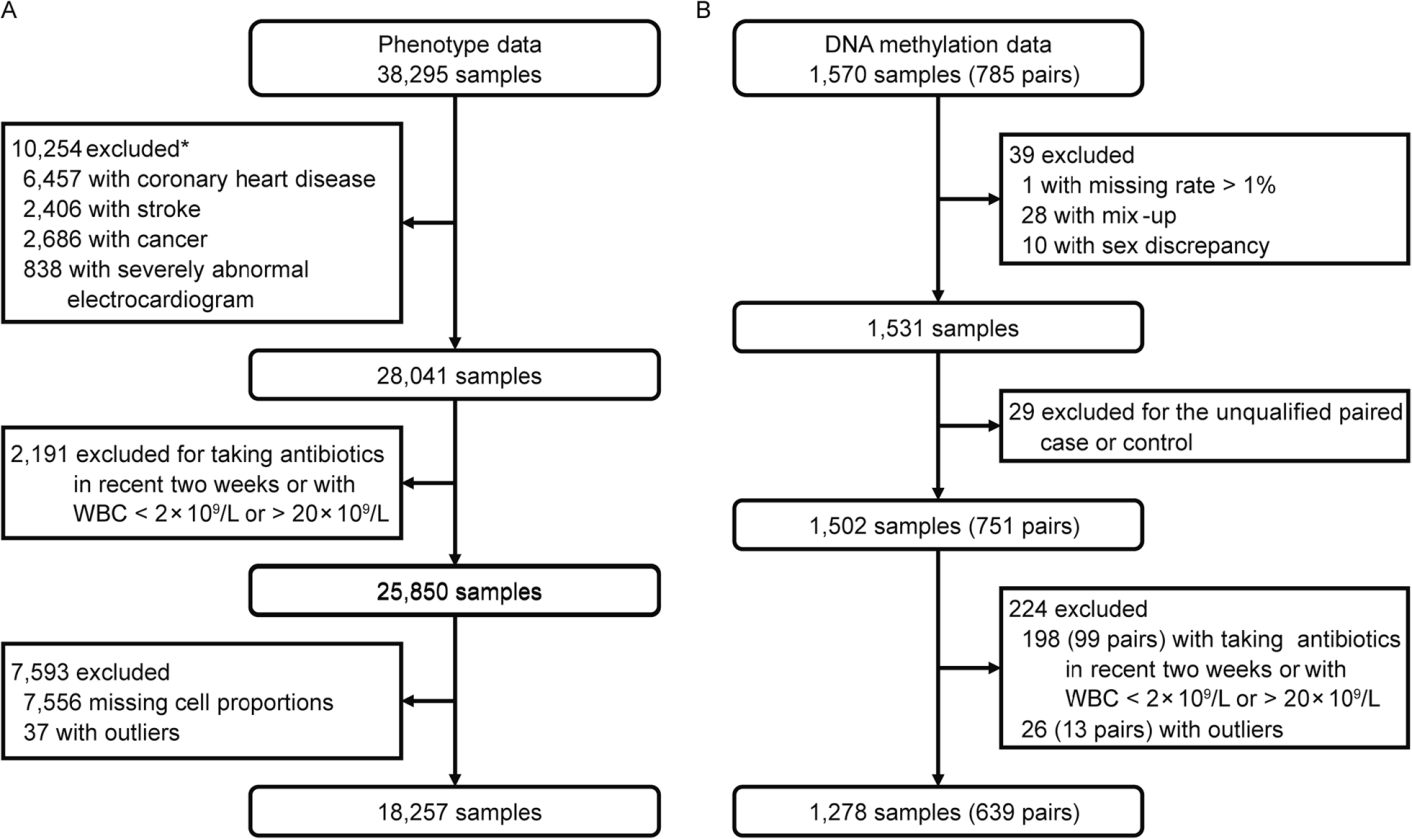
Flowchart of quality controls. **A** Quality controls of phenotype data. **B** Sample quality controls of DNA methylation data. ^*^Some excluded samples meet more than one exclusion criteria

This study was based on the prospective Dongfeng–Tongji cohort, which was launched in 2008 with follow-up every five years. Detailed information of the cohort has been described elsewhere [27, 28]. In this study, we included 38,295 samples, who had participated the first follow-up in 2013. Information on sex, age, height, weight, smoking status, medical history (e.g., hypertension, hyperlipidemia, diabetes, cardiovascular disease, and cancer), and medication in the past two weeks was collected through questionnaires. Blood pressure, percentages of leukocyte subtypes, and biochemical indicators such as lipids and fasting glucose were collected by medical examination. Quality controls are shown in Fig. 1A. We excluded participants if they had prevalent coronary heart disease, stroke, cancer, severely abnormal electrocardiogram or had taken antibiotics within the past two weeks. We also excluded samples with abnormal records of WBC (< 2 × 10^9^/L or > 20 × 10^9^/L), or with missing information or outlier values (> 3 interquartile range (IQR) from the median) of lymphocyte, monocyte, or neutrophil percentages. Finally, a total of 18,257 samples were retained for subsequent analysis.

### Assessment of variables

Incident ACS between the first follow-up in 2013 and December 31, 2018, was identified according to the diagnostic criteria for MI and unstable angina [29, 30]. Body mass index (BMI) was calculated based on height and weight. Obesity was defined as BMI ≥ 28 kg/m^2^ [31]. Hypertension was defined as self-reported physician diagnosis of hypertension, use of anti-hypertension drugs in the last two weeks, systolic blood pressure (SBP) ≥ 140 mmHg, or diastolic blood pressure (DBP) ≥ 90 mmHg [32]. Hyperlipidemia was defined as self-reported physician diagnosis of hyperlipidemia, use of antihyperlipidemic drugs, total cholesterol (TC) ≥ 6.22 mmol/L, high-density lipoprotein cholesterol (HDL-C) < 1.04 mmol/L, low-density lipoprotein cholesterol (LDL-C) ≥ 4.14 mmol/L, or triglyceride (TG) ≥ 2.26 mmol/L [32]. Diabetes was defined as self-reported physician diagnosis of diabetes, use of hypoglycemic drugs or insulin, or fasting glucose (FG) ≥ 7.0 mmol/L [32]. Finally, missing data for BMI were imputed by the sex-specific median values. Missing data on smoking status and disease history were imputed as never smoking and no history of the corresponding disease, respectively.

### Ethics

All participants agreed to participate in the study and signed the written informed consent. The study was approved by the Ethics and Human Subject Committees of Tongji Medical College.

### DNA methylation data

DNA methylation profilings of 785 pairs incident ACS cases and controls were generated from bisulfite-converted genomic DNA using the Infinium HumanMethylationEPIC BeadChip (Illumina Inc, San Diego, CA, USA). Blood samples were collected in the 2013 follow-up of the Dongfeng–Tongji cohort. These 785 incident ACS cases were diagnosed between the first follow-up in 2013 and December 31, 2018. For each incident ACS case, a control, free of CVD and cancer at the time of the case event, was randomly selected according to age (within 1 year), sex, and time of blood sampling (within 6 months).

DNA methylation profiles of 1,570 subjects were processed by the R package *Bigmelon* (v 1.12.0) [33] (Fig. 1B). Probes were filtered if they met the following criteria: (1) missing rate > 5% (*n* = 3960, missing was defined as detection *p* > 0.01 or bead counts < 3); (2) targeting SNP sites (*n* = 59); (3) cross-hybridizing with multiple sites of the genome (*n* = 43,254) [34]; (4) containing SNPs (minor allele frequency > 0.01 in East Asians of 1000 Genomes Project or Singapore Chinese [35]) within 5 bp from 3′ end of the probe (*n* = 26,826) or at the single base extension (*n* = 399); or (5) on sex chromosomes (*n* = 19,627). Samples were excluded if they met the following criteria: (1) missing rate > 1% (*n* = 1, missing was defined as detection *p* > 0.01); (2) mix-up (*n* = 28, mix-up was defined as discordant genotypes at > 10 out of 59 SNPs between the Illumina EPIC array and our existing imputed GWAS array data); and (3) sex discrepancy (*n* = 10, sex discrepancy was defined as the inconsistence between the self-reported and the inferred sex from DNA methylation profiles). Paired cases or controls were excluded if one failed to pass the quality control. We inferred SNP genotypes and sex from the EPIC array by the R package *ewastools* (v1.7) [36] and normalized the data using the *adjustedDasen* function in *watermelon* (v2.2.0) [37]. Next, we used the *Combat* function in the *SVA* package (v3.34.0) [38] to correct for batch effects of plates, with sex, age, smoking status, and BMI as the protected variables. We retained 777,513 probes and 751 pairs of incident ACS case–control samples. For 751 pairs, 99 pairs with antibiotics use within two weeks prior to blood collection or with WBC < 2 × 10^9^/L or > 20 × 10^9^/L, and 13 pairs with outlier values in immune cell proportions were excluded. Finally, a total of 1278 participants were retained for subsequent analysis.

### Immune cell composition estimations

To estimate immune cell composition in peripheral blood, we applied constrained projection, which is a reference-based algorithm implemented in *Bigmelon* [33]. We used the default parameter settings of the *estimateCellCounts.gds* function in *Bigmelon*, except that we changed the reference to FlowSorted.Blood.EPIC [39]. The approach could estimate the proportions of six immune cells, including CD8^+^ T cells, CD4^+^ T cells, B cells, NK cells, monocytes, and neutrophils. To compare with cell proportions derived from routine blood tests, the proportions of CD8^+^ T cells, CD4^+^ T cells, B cells, and NK cells were summed up to estimate the lymphocyte proportion.

### Statistical analysis

Demographic characteristics of participants were presented as mean (standard deviation, SD) for age, median (interquartile range, IQR) for other continuous variables, and counts (percentages) for categorical variables. Difference between groups were compared by t test for age, two-tailed Wilcoxon tests for other continuous variables, and chi-square tests for categorical variables. To assess the accuracy of the estimated immune cell proportions, we compared them with the observed cell proportions derived from routine blood tests using Spearman’s correlation based on 1076 individuals with immune cell composition available from routine blood tests.

For multivariable linear regression analysis, we transformed the proportions of leukocyte subtypes by rank normalization. To compare the effects of ACS risk factors on different immune cell proportions, we transformed the proportions of leukocyte subtypes by arcsine square root transformation (Additional file 1: Fig. S1) followed by standardization. We tested associations between immune cell composition and risk factors of ACS using a multivariable linear regression model:

$$y={\varvec{\upalpha}}\mathbf{w}+{\varvec{\beta}}{\varvec{x}}+\varepsilon ,$$

where $\mathrm{y}$ represents each standardized cell-type proportion (observed or estimated), $\mathbf{w}$ denotes a vector of covariates, including age and hospital, and ${\varvec{\upalpha}}$ is a vector of corresponding coefficients, ${\varvec{x}}$ denotes a vector of variables of interest, including sex, current smoking, past smoking, obesity, hypertension, hyperlipidemia, and diabetes, and ${\varvec{\beta}}$ is a vector of corresponding coefficients, and $\varepsilon$ denotes the residual error.

We applied Cox proportional hazard (Cox-PH) models and conditional logistic regression models to investigate the associations of incident ACS with the observed and estimated immune cell proportions, respectively. We adjusted for ACS risk factors in the analyses. Statistical significance was defined as *P* < 0.05. All analyses were performed in R-4.0.4.

### Role of funding source

All funders did not involve in study design, data collection, data analyses, interpretation, writing of the paper, or decision to submit the paper.

## Results

### Demographic characteristics of participants

After quality control by strict exclusion criteria, 18,257 of the 38,295 samples were retained. The mean ages of the retained 7,797 males and 10,460 females were 66.86 (SD = 6.60) and 60.54 (8.31), respectively (Additional file 2: Table S1). By December 31, 2018, there were 1096 incident cases of ACS from 18,257 participants with a median follow-up of 5.42 years. Age, the proportions of male, obesity, hypertension, hyperlipidemia, diabetes, current smoking, and past smoking were higher in the incident ACS group (*P* < 0.05) (Table 1). For the observed immune cell proportions from routine blood tests**,** the proportion of neutrophils was higher in the incident ACS group, while the proportion of lymphocytes was opposite.

**Table 1:** Characteristics of 18,257 participants grouped by incident ACS

Variable*	Incident ACS (*n* = 1096)	Control (*n* = 17,161)	*P*
Male, *n* (%)	589 (53.74)	7,208 (42.00)	3.31 × 10^–14^
Age, years	67.19 ± 7.95	62.99 ± 8.20	3.75 × 10^–58^
Lym	0.298 (0.242–0.357)	0.313 (0.257–0.371)	1.39 × 10^–8^
Mono	0.060 (0.045–0.075)	0.061 (0.046–0.075)	0.097
Neu	0.604 (0.546–0.667)	0.590 (0.530–0.649)	1.15 × 10^–7^
Obesity, *n* (%)	155 (14.14)	1,864 (10.86)	9.41 × 10^–4^
Hypertension, *n* (%)	849 (77.46)	9,898 (57.68)	6.28 × 10^–38^
Hyperlipidemia, *n* (%)	571 (52.10)	6,813 (39.70)	6.67 × 10^–16^
Diabetes, *n* (%)	294 (26.82)	2,934 (17.10)	3.84 × 10^–16^
*Smoking status, n (%)*			
Current smoking	247 (22.54)	2,851 (16.61)	1.67 × 10^–9^
Past smoking	160 (14.60)	1,664 (9.70)	4.78 × 10^–10^

*The proportions of leukocyte subtypes are from blood routine tests. *Lym* lymphocyte proportion, *Mono* monocyte proportion, *Neu* neutrophil proportion

The 1278 individuals with DNA methylation profiles included 630 males and 648 females, with mean ages of 67.22 (5.99) and 62.99 (7.80), respectively (Additional file 2: Table S2). Major risk factors of ACS were enriched in the ACS case group (*P* < 0.05), except for current smoking and past smoking (*P* > 0.05) (Table 2). The non-significant difference may be due to sex matching. For the estimated immune cell proportions from DNA methylation profiles, except for the NK proportion (*P* < 0.05), there were no significant differences in the proportions of neutrophils, lymphocytes, and other lymphocyte subtypes between the ACS case and control groups (*P* > 0.05).

**Table 2:** Characteristics of 1278 participants grouped by ACS cases and controls

Variable^*^	Case (*n* = 639)	Control (*n* = 639)	*P*
Age, years	65.15 ± 7.22	65.00 ± 7.34	0.710
Lym	0.317 (0.270–0.373)	0.321 (0.271–0.372)	0.994
Mono	0.064 (0.050–0.079)	0.063 (0.048–0.080)	0.329
Neu	0.554 (0.501–0.612)	0.555 (0.502–0.607)	0.789
CD8T	0.084 (0.058–0.116)	0.084 (0.055–0.116)	0.835
CD4T	0.100 (0.069–0.133)	0.096 (0.068–0.127)	0.127
B	0.029 (0.017–0.044)	0.028 (0.016–0.041)	0.109
NK	0.092 (0.065–0.126)	0.100 (0.071–0.129)	0.026
Obesity, *n* (%)	92 (14.40)	58 (9.08)	4.13 × 10^–3^
Hypertension, *n* (%)	478 (74.80)	381 (59.62)	1.06 × 10^–8^
Hyperlipidemia, *n* (%)	326 (51.02)	239 (37.40)	1.27 × 10^–6^
Diabetes, *n* (%)	168 (26.29)	118 (18.47)	1.01 × 10^–3^
*Smoking status, n (%)*			
Current smoking	137 (21.44)	115 (18.00)	0.074
Past smoking	85 (13.30)	67 (10.49)	0.075

*The proportions of leukocyte subtypes are those estimated from DNA methylation profiles. *Lym* lymphocyte proportion, *Mono* monocyte proportion, *Neu* neutrophil proportion, *CD8T* CD8^+^ T cell proportion, *CD4T* CD4^+^ T cell proportion, *B* B cell proportion, *NK* natural killer cell proportion

### Evaluation of accuracy of estimated immune cell composition

**Fig. 2:**
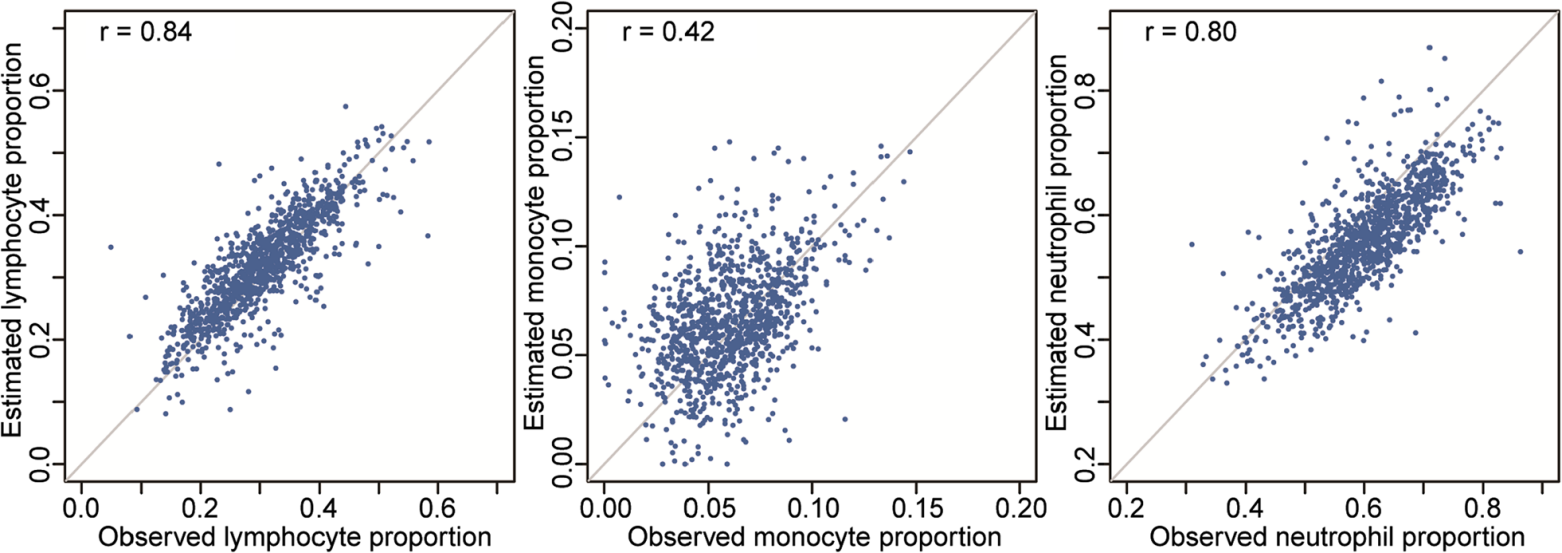
Correlation between the estimated and observed immune cell proportions in 1076 individuals. The estimated immune cell proportions are inferred from DNA methylation profiles by *Bigmelon*, and the observed immune cell proportions are from blood routine tests. Estimated lymphocyte proportion is the sum of proportions of CD8^+^ T cells, CD4^+^ T cells, B cells, and NK cells. The gray lines are diagonal through the origin, and the Spearman’s rank correlation coefficients are displayed on the top left

Among 1076 individuals with both DNA methylation profiles and routine blood tests, Spearman’s correlation coefficients between the estimated and observed proportions of lymphocytes, monocytes, and neutrophils were 0.84, 0.42, and 0.80, respectively (Fig. 2). Overall, the correlations were high, and thus, the immune cell proportions estimated from DNA methylation profiles could reasonably represent those observed from routine blood tests.

### Association of immune cell composition with the risk factors of ACS

**Fig. 3:**
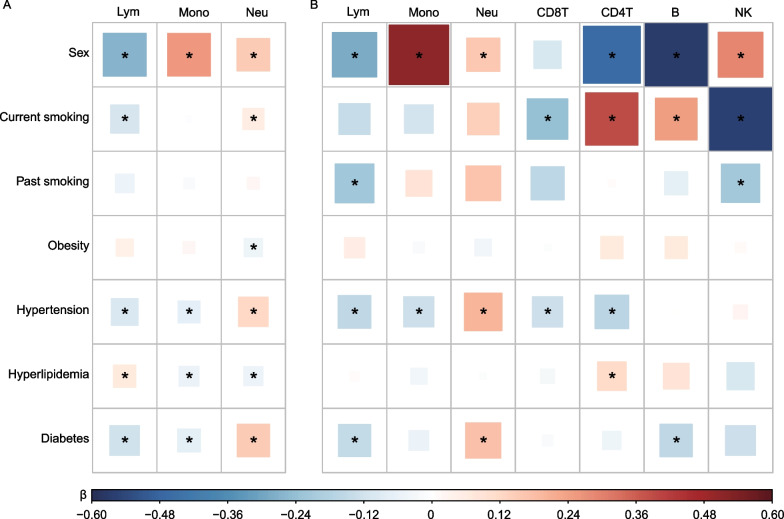
Correlation heatmap of immune cell composition and risk factors of ACS. **A** Association between observed immune cell composition from blood routine tests and risk factors of ACS in 18,257 samples. **B** Association between immune cell composition estimated from DNA methylation profiles and risk factors of ACS in 1278 samples. All observed and estimated immune cell proportions were rank-normalized. The color label denotes the effect size (*β*), the standard deviation changes in the normalized cell proportion for different variable levels of risk factors of ACS. Red indicates that these factors are positively associated with immune cell proportions, while blue indicates an inverse association. The size of the square denotes the effect of factors on each cell proportion, and asterisk suggests significant association (*P* < 0.05). Lym, lymphocyte proportion; Mono, monocyte proportion; Neu, neutrophil proportion; CD8T, CD8^+^ T cell proportion; CD4T, CD4^+^ T cell proportion; B, B cell proportion; and NK, natural killer cell proportion

The proportions of immune cells derived from routine blood tests were associated with sex, current smoking, obesity, hypertension, hyperlipidemia, and diabetes, but not with past smoking (Fig. 3A, Additional file 2: Table S3). Specifically, we observed a lower proportion of lymphocytes and a higher proportion of neutrophils in males, current smokers, and individuals with hypertension or diabetes (*P* < 0.05), whereas the opposite was observed in individuals with hyperlipidemia. In addition, individuals with obesity had a lower proportion of neutrophils (*β* =  − 0.049, 95% CI: − 0.094– − 0.003, *P* = 0.037). For the proportion of monocyte, there was an increase in males (*β* = 0.242, 0.207–0.278, *P* = 1.81 × 10^−40^) and a decline in individuals with hypertension (*β* =  − 0.063, − 0.090– − 0.035, *P* = 8.12 × 10^−6^), hyperlipidemia (*β* =  − 0.048, − 0.075– − 0.022, *P* = 3.91 × 10^−4^), or diabetes (*β* =  − 0.071, − 0.105– − 0.037, *P* = 4.84 × 10^−5^). Among all risk factors, sex had the strongest association with immune cell composition, followed by diabetes.

Associations between immune cell proportions estimated from DNA methylation profiles and the risk factors of ACS are shown in Fig. 3B and Additional file 2: Table S3. For the three main leukocyte subtypes, we only replicated associations with sex and hypertension, and the association of the proportions of lymphocytes and neutrophils with diabetes (*P* < 0.05). Although we failed to replicate other associations identified by the observed immune cell composition, the effect directions of these associations were consistent. For the estimated lymphocyte subtypes, the proportion of CD8^+^ T cells was decreased in current smokers, and hypertensive individuals (*P* < 0.05). The proportion of CD4^+^ T cells decreased in males and hypertensive individuals but increased in current smokers, and individuals with hyperlipidemia (*P* < 0.05). There was a decline in B cell proportion in males and diabetic individuals, and an increase in current smokers (*P* < 0.05). In addition, NK cell proportion was positively associated with sex, and negatively associated with current smoking and past smoking (*P* < 0.05). Among the significant associations, sex and current smoking were the top two risk factors with the greatest effect size on the proportions of lymphocyte subtypes.

### Association of immune cell composition with incident ACS

**Fig. 4:**
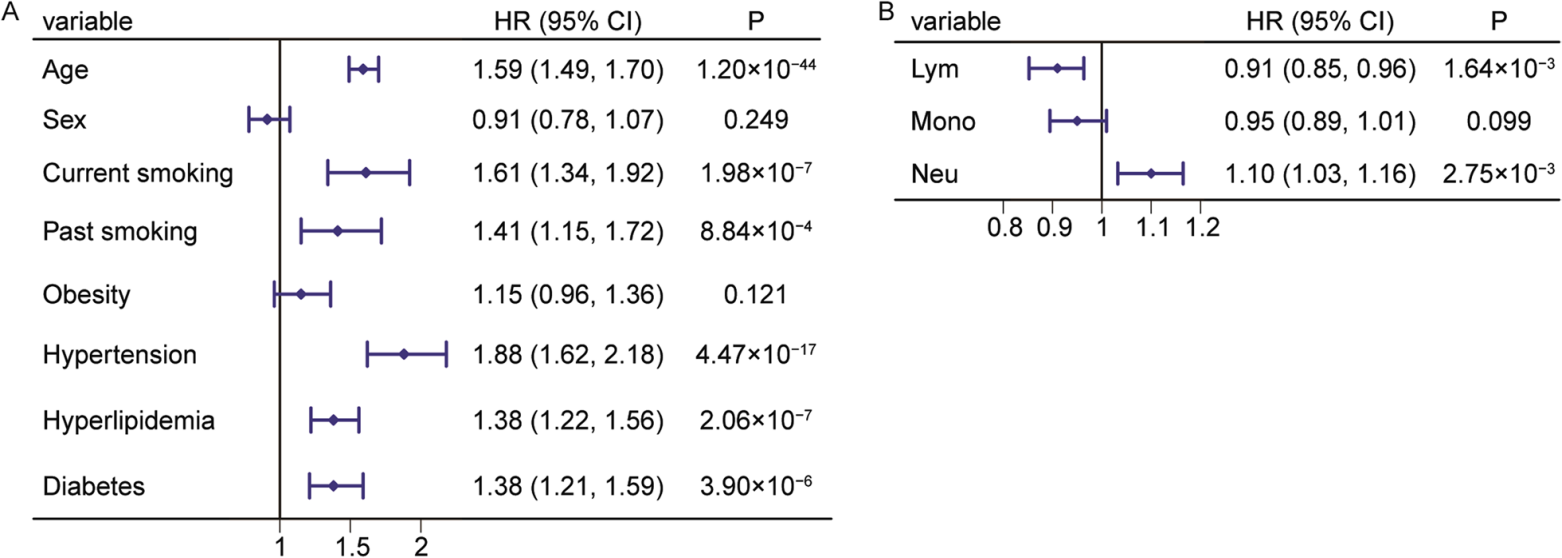
Association of observed immune cell composition from routine blood tests with the incidence of ACS. Association of the risk factors (**A**) and the immune cell proportions from routine blood tests (**B**) with the incidence of ACS in 18,257 samples. For association test between each immune cell composition and the incidence of ACS, the risk factors of ACS were adjusted. Lym, lymphocyte proportion; Mono, monocyte proportion; and Neu, neutrophil proportion

Cox-PH models showed significant associations between incident ACS and these risk factors (*P* < 0.05) except for sex and obesity (Fig. 4A), which was probably due to collinearity. For immune cell-related indicators observed from routine blood tests, Cox-PH models revealed that the hazard ratio (HR) for incident ACS per one SD in lymphocyte proportion was 0.91 (95% CI: 0.85–0.96, *P* = 1.64 × 10^−3^, Fig. 4B) after adjusting for the risk factors of ACS. In addition, the HR of neutrophil proportion was 1.10 (1.03–1.16, *P* = 2.75 × 10^−3^). We found no association between monocyte proportion and incident ACS (*P* > 0.05).

**Fig. 5:**
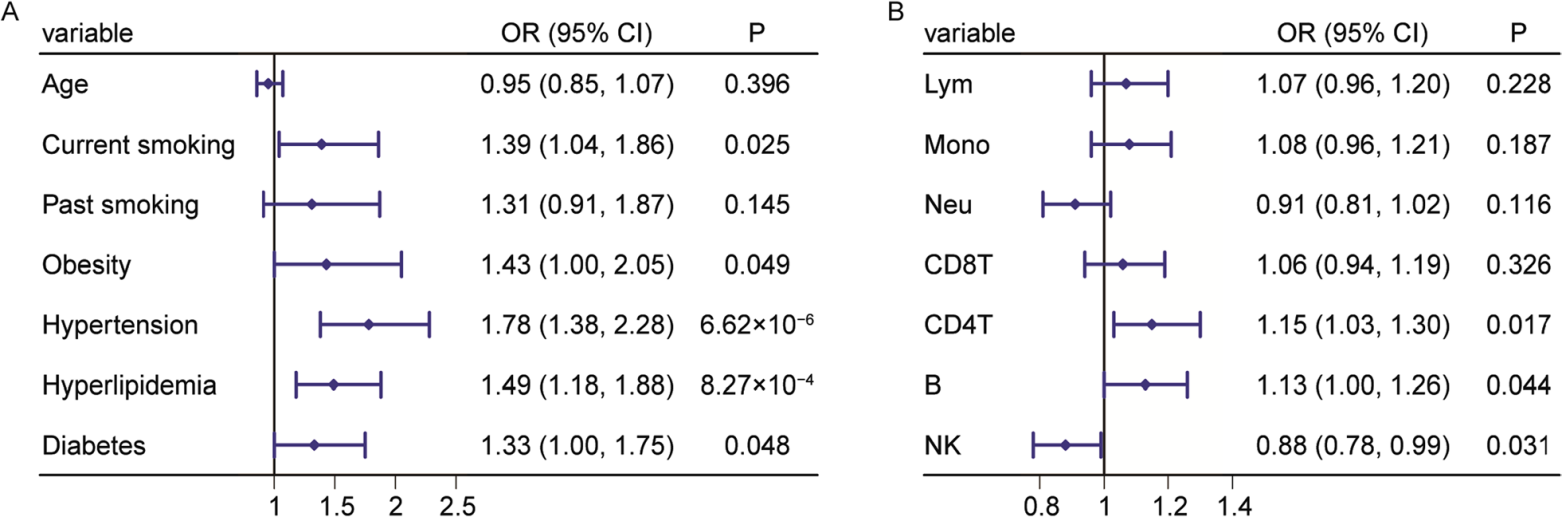
Association of estimated immune cell composition from DNA methylation profiles with the incidence of ACS. Association of the risk factors (**A**) and the immune cell proportions from DNA methylation profiles (**B**) with the incidence of ACS in 1278 samples. For association test between each immune cell composition and the incidence of ACS, the risk factors of ACS were adjusted. Lym, lymphocyte proportion; Mono, monocyte proportion; Neu, neutrophil proportion; CD8T, CD8^+^ T cell proportion; CD4T, CD4^+^ T cell proportion; B, B cell proportion; and NK, natural killer cell proportion

We further investigated the association between estimated immune cell proportions and incident ACS. Conditional logistic regression models showed significant associations between ACS and these risk factors (*P* < 0.05) except for age and past smoking (Fig. 5A), which was probably due to age matching and collinearity or smaller sample size. For immune cell proportions estimated from DNA methylation profiles, we did not observe significant associations between the proportions of lymphocytes, monocytes, and neutrophils and ACS (*P* > 0.05, Fig. 5B) after adjusting for major risk factors of ACS. However, the proportions of lymphocyte subtypes were significantly associated with incident ACS. The adjusted ORs for proportions of CD4^+^ T cells, B cells, and NK cells were 1.15 (1.03–1.30, *P* = 0.017), 1.13 (1.00–1.26, *P* = 0.044), and 0.88 (0.78–0.99, *P* = 0.031), respectively.

## Discussion

In the present study, we analyzed the association between blood immune cell proportions and incident ACS in cohort and nested case–control study. We found that the proportions of lymphocytes and NK cells were negatively associated with incident ACS, while the proportions of neutrophils, CD4^+^ T cells, and B cells were positively associated with incident ACS. In addition, we jointly analyzed the associations between major risk factors of ACS and blood immune cell composition in the Dongfeng–Tongji cohort. We identified that sex and diabetes had the strongest associations with the proportions of lymphocytes, monocytes, and neutrophils. Furthermore, sex and current smoking had the strongest associations with the proportions of lymphocyte subtypes.

Previous studies showed that neutrophil counts and NLR were positively associated with incident ACS [9, 10]. After adjusting for major risk factors of ACS, we found that the proportions of neutrophils and lymphocytes were positively and negatively associated with the incident ACS, respectively. Neutrophils are involved in atherosclerosis and promote the size of plaques [40], and high levels of neutrophils indicate plaque destabilization and rupture-prone lesions [41]. Furthermore, high levels of neutrophils are often associated with poor clinical outcomes in ACS patients [42]. Lymphocytes play a key role in the regulation of inflammation response in atherosclerosis [43]. Uncontrolled immune system activation might increase the apoptosis of lymphocytes [44]. Moreover, a lower proportion of lymphocytes in ACS patients predicts a higher risk of recurrent cardiovascular diseases [45].

The protective role of lymphocyte proportion in ACS might be attributed to NK cells. Despite that Olson et al. [46] did not find an association between NK cell proportion and the incidence of MI, several other studies found a lower proportion of NK cells in ACS patients compared to stable angina patients and healthy controls [47, 48], suggesting a protective role of NK cells in ACS. While NK cells had no effect on atherosclerosis development, they had a proinflammatory effect on atherosclerosis due to chronic inflammation [49]. As a part of innate immune, the decrease or dysfunction of NK cells might increase the susceptibility to atherogenic antigen, inducing chronic inflammation to promote atherosclerosis [50]. The association between NK cells and atherosclerosis and cardiovascular diseases warrants further study.

This study showed the positive association between the proportions of CD4^+^ T cells and B cells and incident ACS. Atherosclerosis, the underlying mechanism of ACS, is characterized by the accumulation of lipids and immune cells [51]. CD4^+^ T cells could proliferate and secrete cytokines to promote atherosclerosis when restimulated with low-density lipoprotein cholesterol (LDL-C) and apolipoprotein B (ApoB) [52, 53]. In addition, single-cell transcriptomics showed that T cells were the most abundant population of immune cells in human carotid atherosclerotic plaques [54], suggesting that CD4^+^ T cells were involved in atherosclerosis. B cells include different B cell subtypes, such as follicular B cells and B1 cells, which have proatherogenic and antiatherogenic effect, respectively [55]. Further studies on the association between B cell subtypes and atherosclerosis might lead to new treatment strategies for cardiovascular diseases.

Our study showed that males had a lower proportion of lymphocytes and higher proportions of monocytes and neutrophils, which supported the sex differences in immune cell composition in the elderly population [20, 21]. Furthermore, the proportions of CD4^+^ T cells and B cells decreased in males, while NK cell proportion increased. Consistently, Márquez et al. [56] also observed lower proportions of CD4^+^ T cells and B cells in males older than 65. In current smokers, the proportion of lymphocytes decreased and the proportion of neutrophils increased, consistent with previous studies in European populations [13, 22]. Based on the estimated lymphocyte subtypes, the decrease in lymphocytes was mainly attributed to NK cells and CD8^+^ T cells, in line with a study based on 22 monozygotic twins of European ancestry [14]. Similar to previous studies [57–59], we found no associations between immune cell composition and past smoking, except that the association between the proportion of NK cells and past smoking needed further validation. Moreover, comparing to current smoking, the effect size of past smoking on incident ACS decreased, suggesting that quitting smoking could restore the homeostasis of immune cell composition and prevent smoking-related diseases. In addition, we found that individuals with diabetes had a higher proportion of neutrophils and a lower proportion of lymphocytes. Consistently, previous studies have shown that a higher neutrophil-to-lymphocyte ratio (NLR) is associated with diabetes [60, 61]. We found that the reduced lymphocyte proportion might be primarily attributed to reduced B cells. Previous studies have observed that B cell subsets, but not B cells, are associated with diabetes [62, 63]. The inconsistency is probably due to differences in sample size and population, suggesting that further studies are needed.

There are several strengths in this study. Firstly, we jointly analyzed the association of immune cell composition with the risk factors of ACS in a large Chinese population, which could provide greater statistical power due to its large sample size. Secondly, we showed the value of immune cell proportions in the assessment of the risk of incident ACS, which might be due to the mediating role of immune cell proportions in the association between ACS and its risk factors. Thirdly, our study took full advantage of DNA methylation profiles to comprehensively study the effects of the proportions of three main leukocyte subtypes and lymphocyte subtypes on the incidence of ACS. However, several limitations also exist. Firstly, we did not obtain lymphocyte subtype percentages from flow cytometry to further evaluate the performance of estimated lymphocyte subtypes. Nevertheless, previous studies have suggested that immune cell composition estimated from DNA methylation is accurate and reliable [25, 64]. Meanwhile, the correlation of monocyte proportion between the estimated and observed was moderate. Cell-specific DNA methylation reference data from the Chinese populations or novel cell-type deconvolution methods will help improve the estimation of cell-type proportions. Secondly, the immune cell proportions might change during the follow-up such that a single-time measurement might not be representative. Nevertheless, a previous study showed that the 5-year changes in leukocyte subtypes counts were not associated with incident ACS [9]. Thirdly, our study population was primarily retired workers, such that the applicability of the results to the young population needs further investigation.

## Conclusions

The current study showed that sex, diabetes, and current smoking were significantly associated with the immune cell proportions, suggesting that controlling for these factors might help predict disease progression more precisely. In particular, neutrophil proportion showed a risk effect on the incidence of ACS, while lymphocyte proportion showed a protective effect, which might be attributed to NK cells. The immune cell proportions might contribute to the risk of ACS.

## Supplementary Information

**Additional file 1. Figure S1. Histograms of immune cell proportions after arcsine square root transformation.** Immune cell composition observed from routine blood tests (A) and estimated from DNA methylation profiles (B). Lym, lymphocyte proportion; Mono, monocyte proportion; Neu, neutrophil proportion; CD8T, CD8^+^ T cell proportion; CD4T, CD4^+^ T cell proportion; B, B cell proportion; and NK, natural killer cell proportion.

**Additional file 2. Table S3.** Association between immune cell composition and risk factors of ACS.

## Data Availability

Data are available on request from the corresponding author upon reasonable request.
