# Supplementary material for: Association of immune cell composition with the risk factors and incidence of acute coronary syndrome
Source: Clin Epigenetics. 2023 Jul 17;15:115. doi: 10.1186/s13148-023-01527-4 (PMC10353119; doi:10.1186/s13148-023-01527-4)
Supplement: Supplementary file 1 — Additional file 1. Figure S1. Histograms of immune cell proportions after arcsine square root transformation. Immune cell composition observed from routine blood tests (A) and estimated from DNA methylation profiles (B). Lym, lymphocyte proportion; Mono, monocyte proportion; Neu, neutrophil proportion; CD8T, CD8+ T cell proportion; CD4T, CD4+ T cell proportion; B, B cell proportion; and NK, natural killer cell proportion. [file 13148_2023_1527_MOESM1_ESM.docx]

**Supplementary Materials for**

**Association of immune cell composition with the risk factors and incidence of acute coronary syndrome**

Xian Shi^1^, Minghan Qu^1^, Yi Jiang^1^, Ziwei Zhu^1^, Chengguqiu Dai^1^, Minghui Jiang^1^, Lin Ding^1^, Yu Yan^1^, Chaolong Wang^1^, Xiaomin Zhang^2^, Shanshan Cheng^1^*, Xingjie Hao^1^*


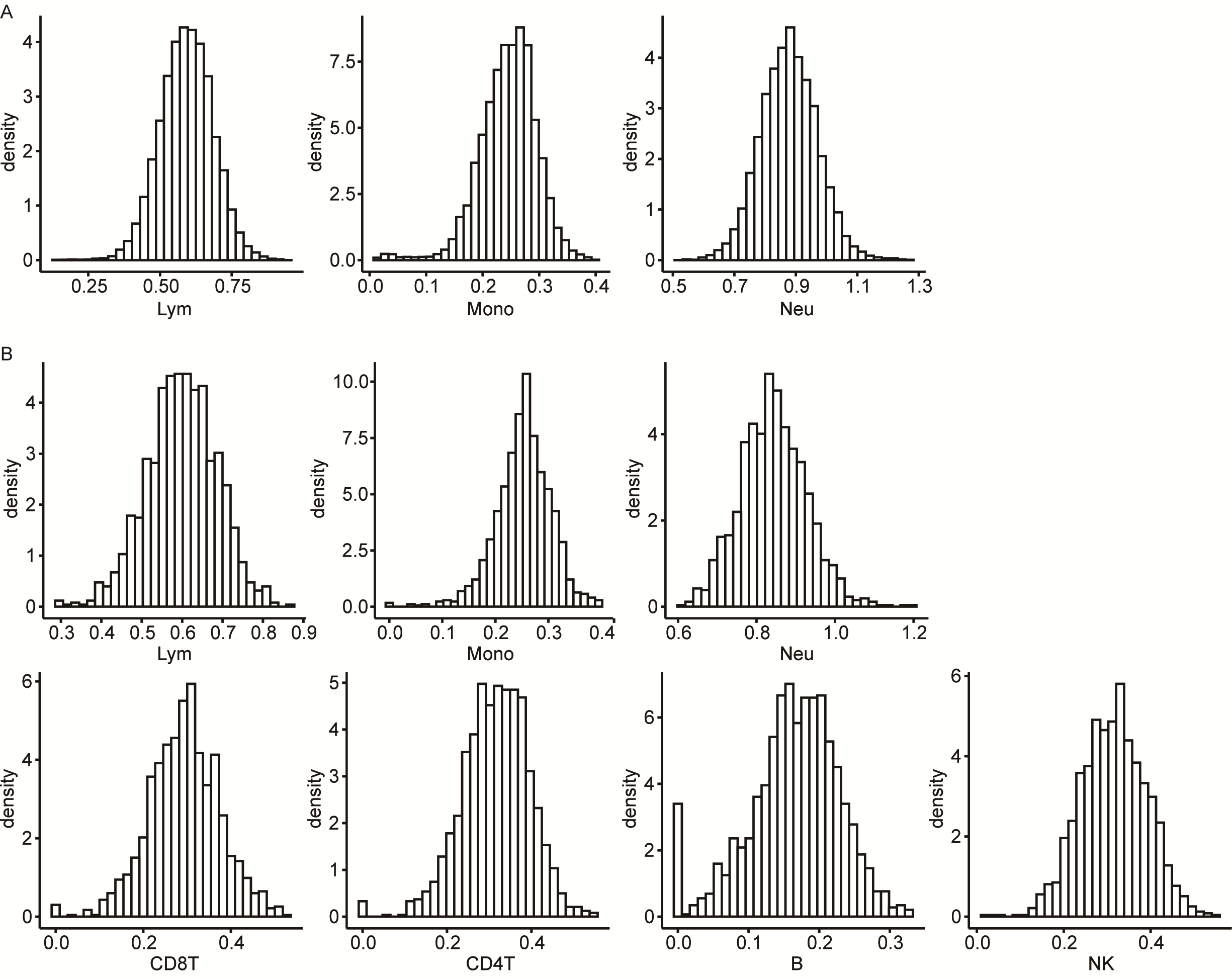


**Figure S1. Histograms of immune cell proportions after arcsine square root transformation.** Immune cell composition observed from routine blood tests (A) and estimated from DNA methylation profiles (B). Lym, lymphocyte proportion; Mono, monocyte proportion; Neu, neutrophil proportion; CD8T, CD8^+^ T cell proportion; CD4T, CD4^+^ T cell proportion; B, B cell proportion; and NK, natural killer cell proportion.
